# Pathological fracture of the femur in Alagille syndrome that was treated with low-intensity pulsed ultrasound stimulation and an Ilizarov ring fixator: a case report

**DOI:** 10.1186/1471-2474-15-225

**Published:** 2014-07-08

**Authors:** Koji Nozaka, Yoichi Shimada, Naohisa Miyakoshi, Shin Yamada, Yuji Kasukawa, Atsuko Noguchi

**Affiliations:** 1 Department of Orthopedic Surgery, Akita University Graduate School of Medicine, Hondo, Akita 010-8543, Japan; 2 Department of Pediatrics, Akita University Graduate School of Medicine, Hondo, Akita 010-8543, Japan

**Keywords:** Alagille syndrome, Low-intensity pulsed ultrasound stimulation (LIPUS), Ilizarov ring fixator

## Abstract

**Background:**

Alagille syndrome is a multisystem disorder, which is characterized by hypoplasia of the intrahepatic bile ducts, malformations of the cardiovascular system, eyes, and vertebral column, and abnormal facies. Several of the characteristics of Alagille syndrome may result in an especially high risk of fracture. The majority of patients suffer from chronic cholestasis, which can have a variety of adverse effects on bone metabolism. In Alagille syndrome, fractures primarily occur in the lower limb long bones in the absence of significant trauma.

**Case presentation:**

A 9-year-old Japanese girl with Alagille syndrome was admitted to our institution with marked hyperbilirubinemia and a pathological fracture of the femur. She had been diagnosed with biliary atresia at the age of 1 month and treated with surgical bile duct reconstruction, vitamins D and K, and ursodeoxycholic acid. However, her liver dysfunction and hyperbilirubinemia worsened. The pathological fracture of the femur was treated with low-intensity pulsed ultrasound stimulation (LIPUS) and an Ilizarov ring fixator. Seventy-four days after surgery, the patient had anatomically and functionally recovered. There was no leg-length discrepancy and no angular malalignment of the lower extremities as measured clinically and radiographically. The range of motion of the hip, knee, and ankle of the patient’s operative leg matched the range of motion in the nonoperative leg.

**Conclusion:**

To the best of our knowledge, there are no reports on use of the Ilizarov frame and LIPUS in diaphyseal femoral fractures in Alagille syndrome. This case report provides evidence that this procedure is successful for managing such diaphyseal fractures in Alagille syndrome.

## Background

Alagille syndrome is a multisystem disorder, which is characterized by hypoplasia of the intrahepatic bile ducts, malformations of the cardiovascular system, eyes, and vertebral column, and abnormal facies [1]. Major clinical features of this syndrome include jaundice, cholestasis, and congenital heart disease with peripheral pulmonary artery stenosis. Alagille syndrome is caused by mutations in the *JAGGED1* gene in the overwhelming majority of cases, and by mutations in the *NOTCH2* gene in 1–2% of cases [2-4]. A genetic test was not performed in our case because the diagnosis of Alagille syndrome was already established based on the presence of distinct clinical features.

Several of the characteristics of Alagille syndrome may result in patients having an especially high risk of fracture. The majority of patients suffer from chronic cholestasis [5], which can have a variety of adverse effects on bone metabolism. Hyperbilirubinemia inhibits osteoblast proliferation and induces osteoporosis. Most importantly, cholestasis leads to a deficiency of intestinal bile acids. This deficiency ultimately interferes with the absorption of vitamins and minerals that are critical to bone development, including calcium, vitamin D, and vitamin K. Management of a pathological lower extremity fracture in a child with Alagille syndrome can sometimes be a challenging problem in the orthopedic field. There are few reports of pathological fractures of the femur in Alagille syndrome. Case studies have documented multiple recurrent fractures in some patients [6] and poor healing and/or marked post-fractural deformities in others [7,8]. We report a case of a 9-year-old girl with marked hyperbilirubinemia from birth who presented with a pathological fracture of the femoral shaft.

## Case presentation

**Figure 1:**
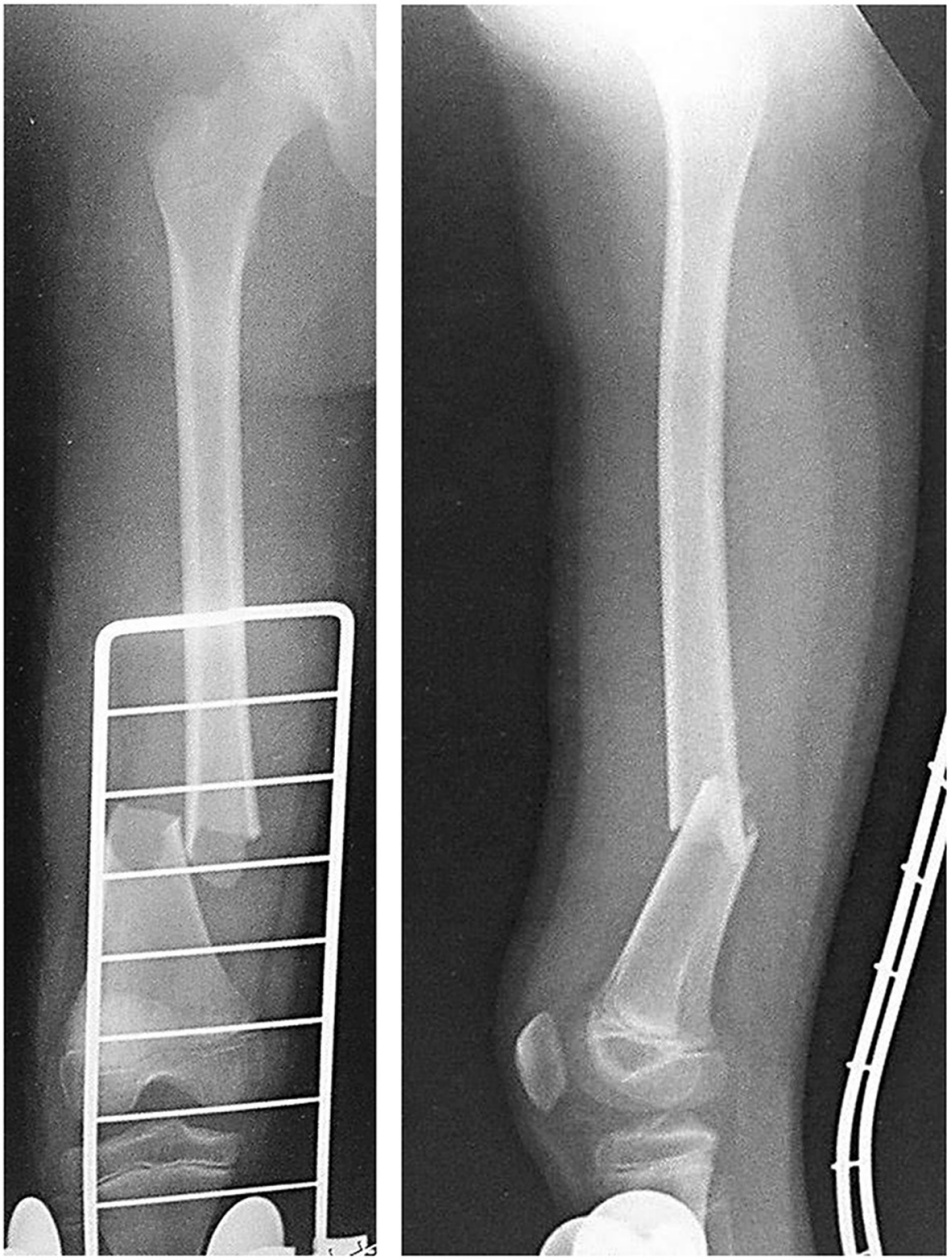
**Preoperative radiographs showing a noncomminuted fracture with a lateral spike.** A right femoral diaphyseal fracture (Orthopaedic Trauma Association classification: 32–A3.2) is shown.

**Figure 2:**
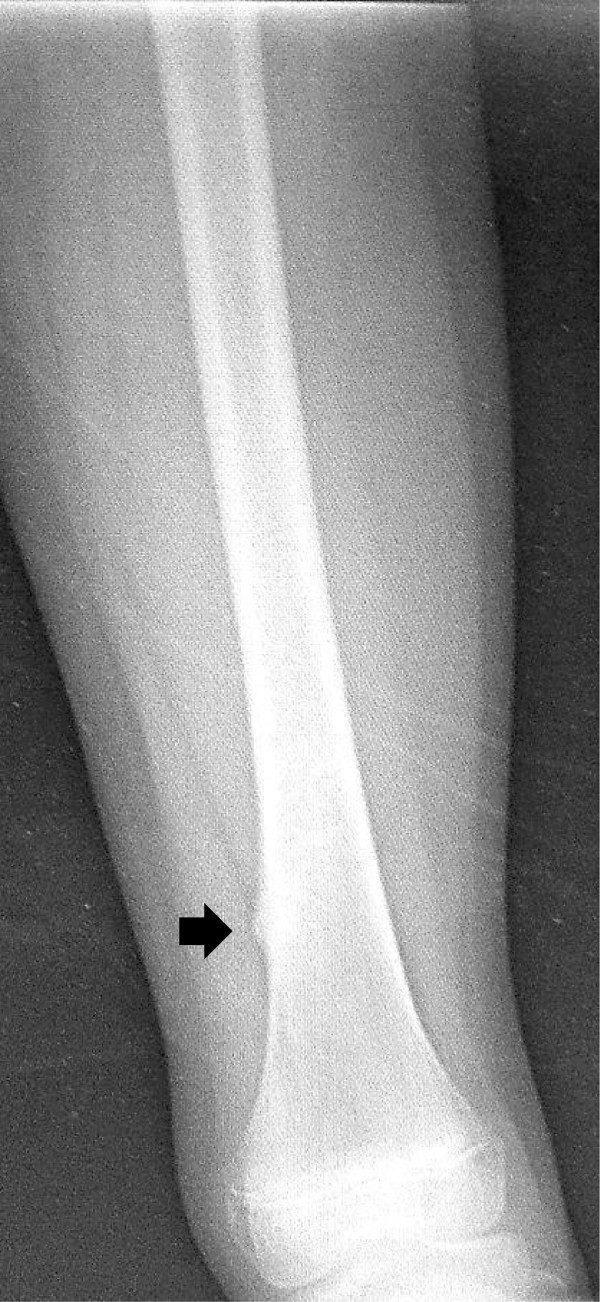
Admission radiograph showing beaking and cortical thickening (arrow) in the left femur.

**Figure 3:**
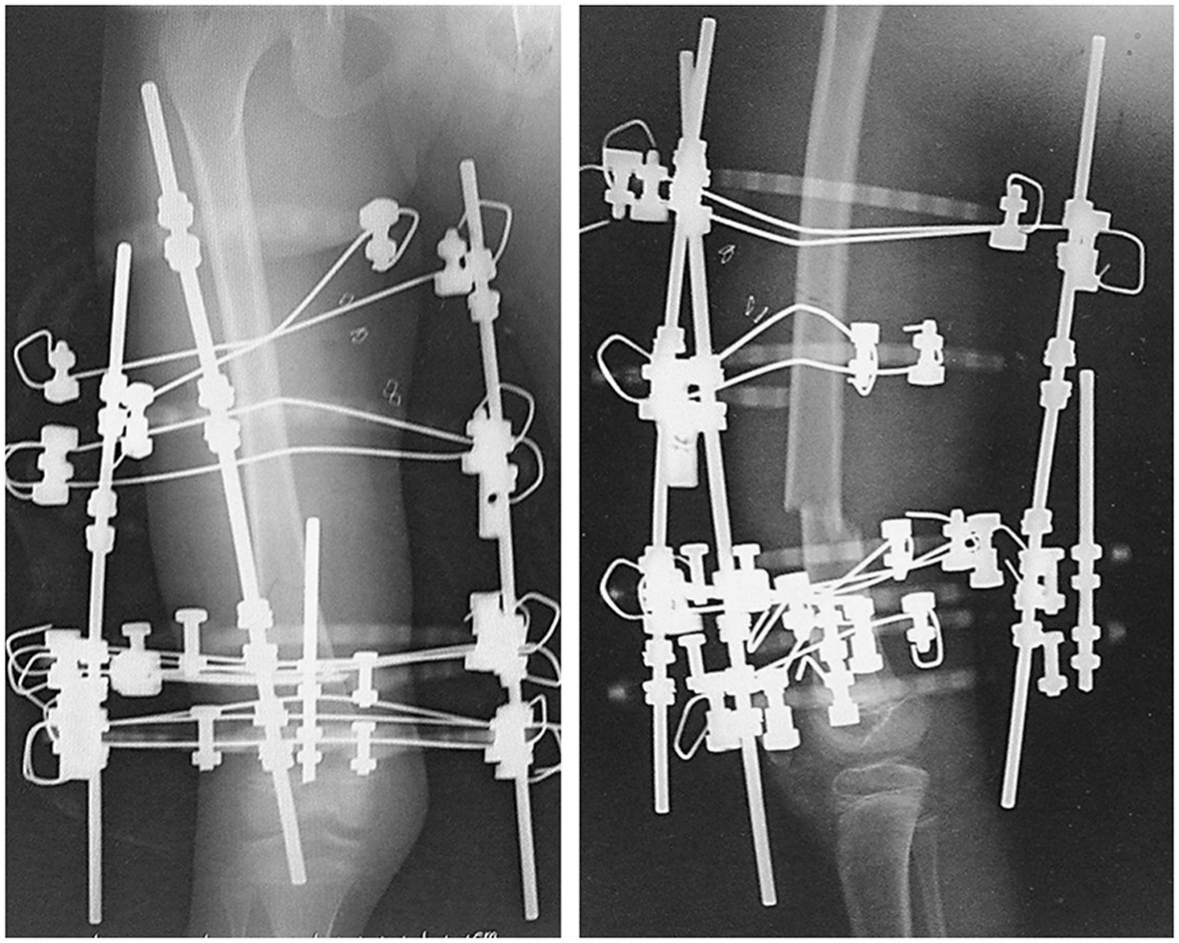
Postoperative radiologic image of the femoral diaphyseal fracture that was treated using an Ilizarov ring fixator.

**Figure 4:**
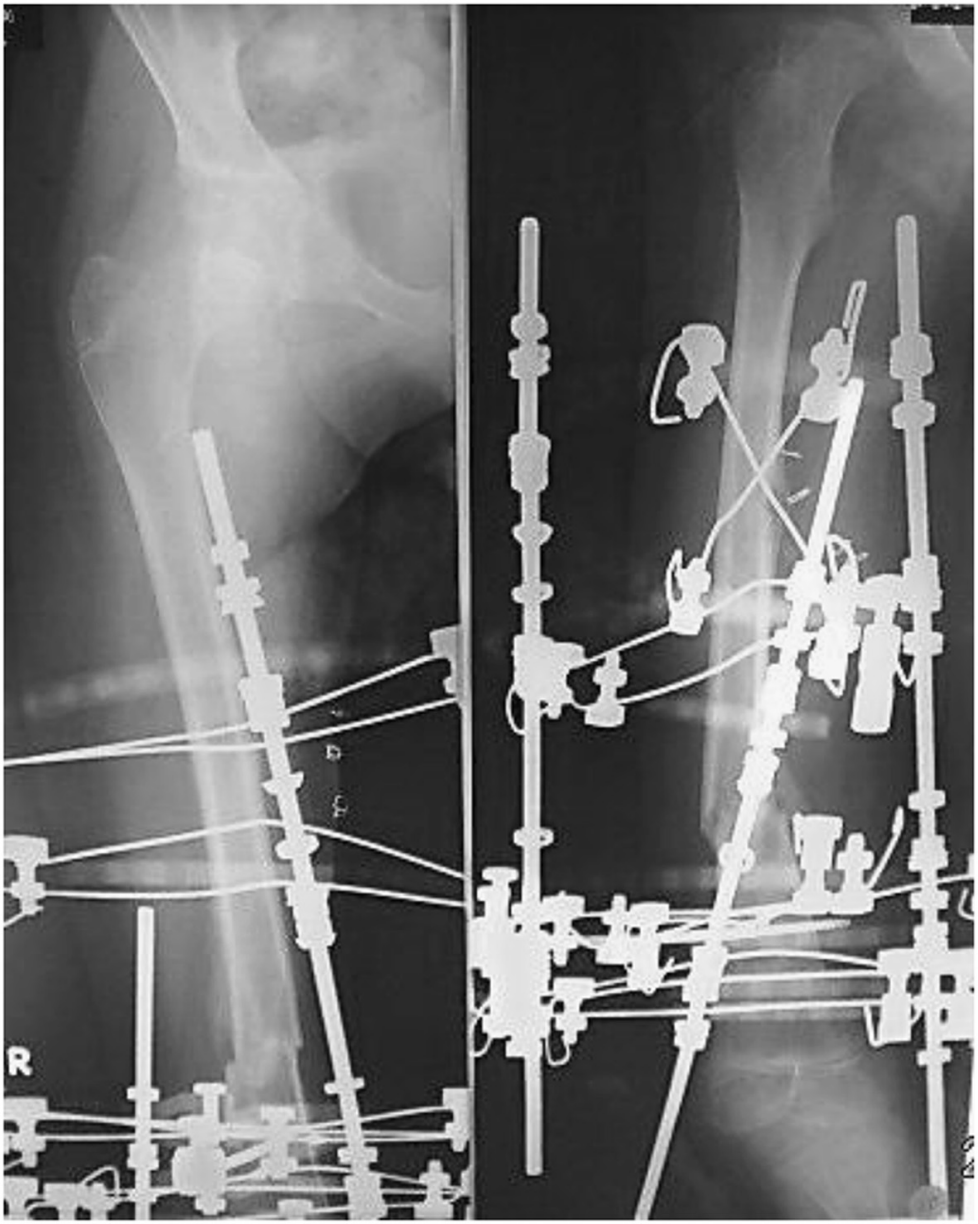
Radiographs show healing of the fracture at 53 days postoperatively.

**Figure 5:**
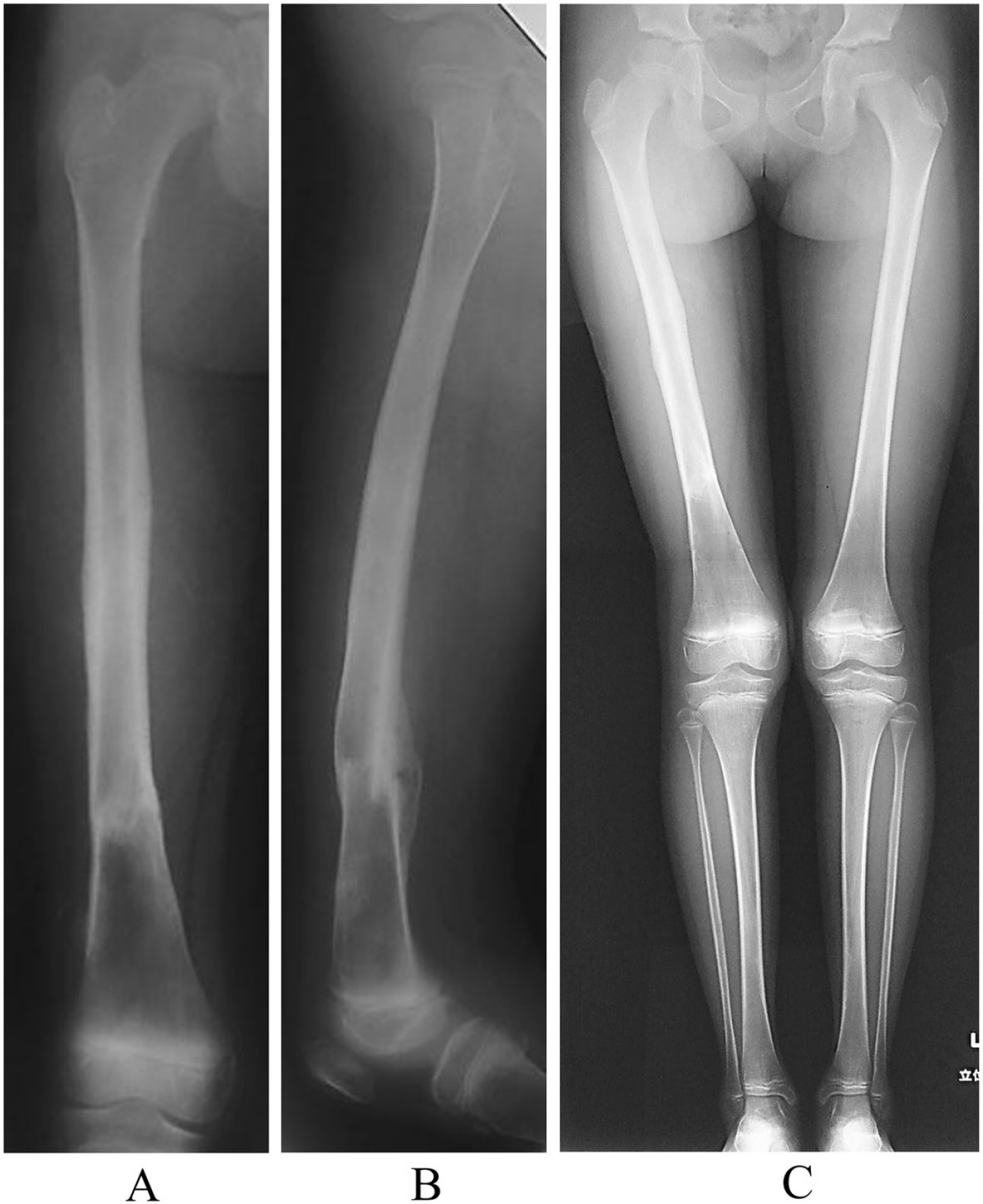
**Radiographs show healing of the fracture at 2 years postoperatively. A:** AP **B:** lateral **C:** Radiographs show no leg-length discrepancy and no angular malalignment of the lower extremities.

**Figure 6:**
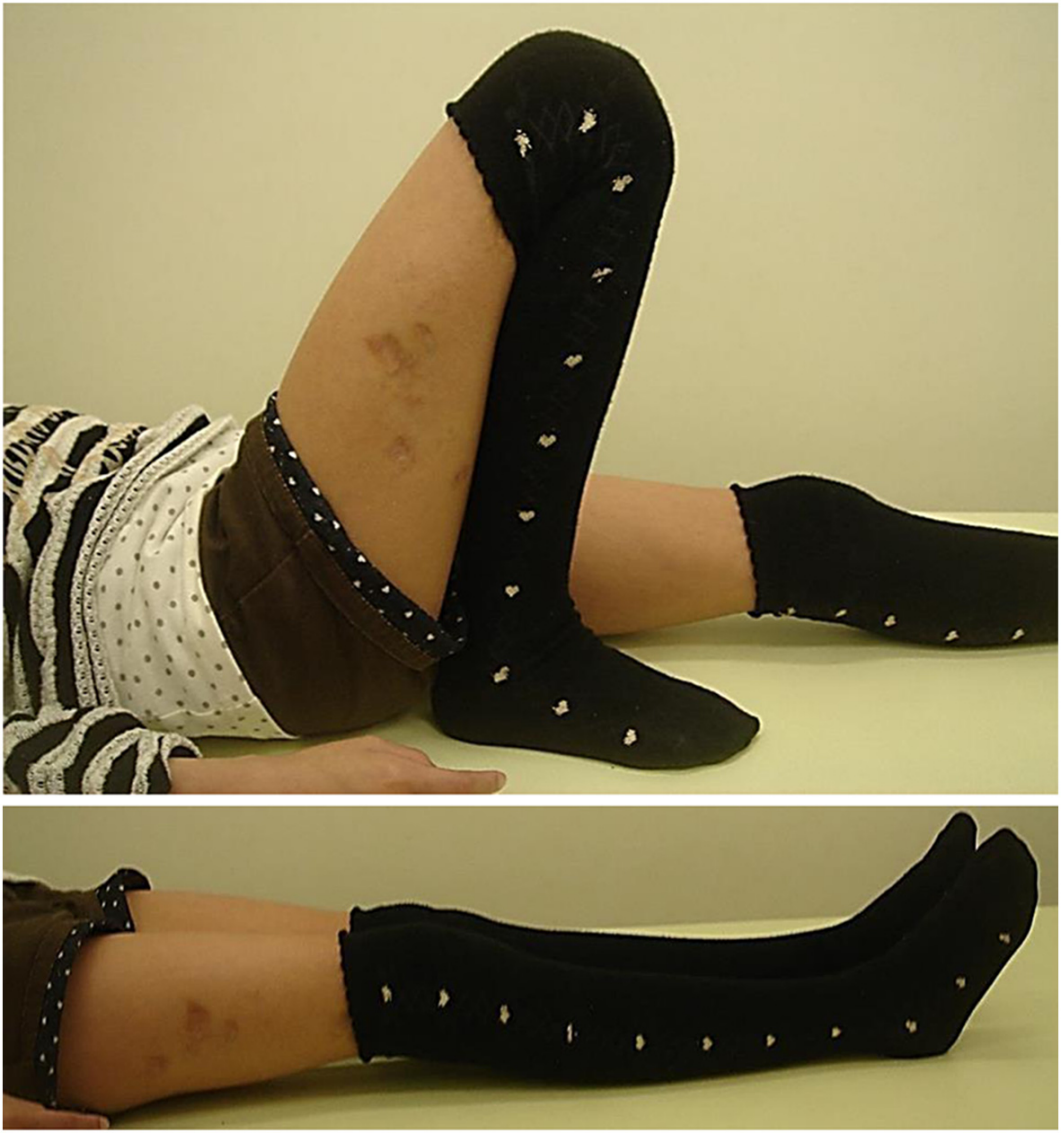
The range of motion in the patient’s operative leg has returned to that of the nonoperative leg.

A 9-year-old girl with Alagille syndrome was referred to our hospital. She had been diagnosed with biliary atresia at the age of 1 month and treated with surgical bile duct reconstruction, vitamins D and K, and ursodeoxycholic acid. However, her liver dysfunction and hyperbilirubinemia worsened. When she was running during physical education, she suddenly felt an acute pain in her right knee. She could not walk and was taken to the emergency department of another hospital. She was found to have a sustained pathological fracture of the right femoral shaft and was treated with skeletal traction. However, repositioning the fractured bone was difficult. Because of her low weight (19 kg), application of skeletal traction with a heavy weight was difficult. On examination, she was malnourished with stunted growth (height: 126 cm, < 3rd centile; weight: 19 kg, < 3rd centile). She had most of the features of Alagille syndrome, including a characteristic face, mild peripheral pulmonary artery stenosis, butterfly vertebrae, posterior embryotoxon, and hyperbilirubinemia. Blood tests revealed anemia (hemoglobin, 8.3 mg/dL) and liver dysfunction with high serum aspartate transaminase (186 U), alanine aminotransferase (253 U), gamma-glutamyl-transpeptidase (1445 IU/L), serum total cholesterol (23.5 mmol/L), and serum alkaline phosphatase (3546 U) levels, as well as hyperbilirubinemia (218.9 μmol/L). Radiographs showed a left femoral shaft fracture (Orthopaedic Trauma Association classification: 32–A3.2) (Figure 1). Elastic nailing was considered; however, because of her narrow intramedullary canal, this was judged to not be a viable fixation method. Furthermore, we wanted to prevent increased bleeding caused by use of a locking plate because of the anemia. The left femur was osteoporotic, with beaking and cortical thickening (Figure 2). Therefore, there appeared to be a risk of pathological fracture of the left femur. We decided to use a closed indirect reduction technique with an Ilizarov ring fixator and to decrease bleeding. One day after admission to our institute, Ilizarov ring fixator surgery was performed with the patient under general anesthesia in the supine position without a thigh tourniquet. For the Ilizarov technique, a closed indirect reduction technique was performed under image guidance, by first using ligamentotaxis to compress the fracture ends (Figure 3). Repositioning was simple and complete. There was no need to open the fracture site, fixation was stable, and the growth plate was preserved. The tota1 operative time was 69 minutes. The hemog1obin concentration decreased from 8.3 mg/dL preoperative1y to 8.1 mg/dL the next day. This patient was not transfused. Immediately after surgery, treatment with a low-intensity pulsed ultrasound stimulation (LIPUS) device (SAFHS 2000, Exogen, Inc., Piscataway, NJ) was started for 20 min/day in September 2000. This device had a frequency of 1.5 MHz, a signal burst width of 200 microseconds, a signal repetition frequency of 1 kHz, and an intensity of 30 mW/cm^2^. There was no need for additional external immobilization. Physical therapy involving walking with weight-bearing on the operated leg was started immediately after surgery. The patient could walk without any support 1 week later. The hospital stay was 14 days. The patient was well after being discharged from hospital and enjoying school life with the frame. Use of LIPUS was continued, and the patient was allowed to walk without crutches. Radiographs showed healing of the fracture at 53 days postoperatively (Figure 4). In such cases, before actually removing the frame, the patient may be allowed full weight-bearing, in which all the uprights connecting the proximal and distal segments of the bone are disconnected, and the patient is asked to use the limb in a functional manner with weight-bearing for the lower limb for 3 weeks. This was performed in our case. Seventy-four days postoperatively, the frame was removed, and the patient had anatomically and functionally recovered. Two years postoperatively, there was no leg-length discrepancy and no angular malalignment of the lower extremities as determined clinically and radiographically. Furthermore, 2 years postoperatively, the range of motion of the hip, knee, and ankle of the patient’s operative leg matched the range of motion in the nonoperative leg (Figures 5 and 6).

## Discussion

Alagille syndrome is rare, occurring in 1 in 70,000 births, and affects both sexes equally [4]. Alagille syndrome is autosomally dominant with a low penetrance and highly variable expression. The Alagille gene has been identified in the 20pl2 region. This syndrome manifests as a multisystem disorder involving the liver, heart, eyes, face, and skeleton [1,8]. One of the manifestations of Alagille syndrome is hyperbilirubinemia caused by cholestasis secondary to a paucity of interlobular bile ducts. Patients with chronic liver disease have an increased prevalence of osteoporosis because of calcium malabsorption caused by low levels of 25-hydroxy vitamin D3 and hyperbilirubinemia. Nonetheless, the underlying mechanism causing osteoporosis secondary to hyperbilirubinemia remains unclear. Patients with primary biliary cirrhosis and osteoporosis have higher serum bilirubin levels than those without osteoporosis. A high serum bilirubin level is independently associated with increased bone loss at the femur in patients with chronic liver disease. Exposure to excessive levels of bilirubin inhibits the proliferation of osteoblasts in cell cultures. Despite these findings, studies investigating the association between bilirubin levels and osteoclast function are lacking [8]. In the present patient, long-term hyperbilirubinemia was associated with systemic osteoporosis.

Lin et al. [9] reported that, in patients with Alagille syndrome, fracture lesions deteriorated despite treatment, and all of the patients eventually succumbed. Many previous reports have described the management of diaphyseal femoral fractures in normal children. However, no clear consensus has been reached on the best way to treat diaphyseal femoral fractures in normal children [10-12]. Kapukaya et al. [13] reported that external fixation in closed femoral shaft fractures in children could be a rational alternative mode of therapy because it has some advantages and can be easily removed without undergoing a second round of anesthesia. The rationale for this technique is immediate weight-bearing.

Many studies regarding the effects of LIPUS on bone have been reported, including an acceleration of endchondral ossification of the callus at the fracture site [14], an increase in aggrecan messenger RNA levels and proteoglycan synthesis in chondrocyte cultures [15], and modulation of transforming growth factor formation and adenylate cyclase production in osteoblasts [16]. In addition, an increase in insulin-like growth factor in bone marrow-derived stromal cells [17] and an increase in prostaglandin E2 production in osteoblasts have been observed [18]. Recently, LIPUS was used for treatment of Charcot joint and leg lengthening [19-21]. In Alagille syndrome, deficiency of intestinal bile acids ultimately interferes with the absorption of vitamin D, and failure of endochondral ossification is generally associated with the disturbance of absorption of vitamin D. However, the underlying mechanism causing delayed union in Alagille syndrome remains unclear. Therefore, we consider that LIPUS might promote enhancement of endochondral ossification in femoral shaft fracture healing in Alagille syndrome. The good clinical healing in the present case indicates that these mechanisms are induced by LIPUS, and that its effects are sufficient for healing of fractures associated with this condition.

To the best of our knowledge, no studies have reported use of the Ilizarov frame and LIPUS in diaphyseal femoral fractures in Alagille syndrome. The rationale for this technique is immediate weight-bearing, good knee and ankle motion, and a high rate of a successful union. Furthermore, the reported average time that fixators are removed is approximately 55 (range, 38–79) days [13]. Following the original principles of the Ilizarov technique, the closed indirect reduction technique was used under image guidance, first using ligamentotaxis to compress the fracture ends [22]. Obtaining perfect reduction and absolute compression of the fracture ends were not difficult. Fracture healing occurred by secondary intention and callus formation. We did not experience wire cut through, especially in the presence of pin-tract infection. The early union in this case, despite having Alagille syndrome, might be attributed to the initial closed reduction. Early ambulation and immediate weight-bearing may improve limb circulation and enhance the healing process, based on the fact that the speed of fracture healing is usually proportional to the amount of available circulation to and between the fragments [23]. One of the most important advantages of using this technique is the excellent recorded knee and ankle range of motion within a short time from frame removal. Active and passive movements of both joints were allowed and encouraged along the entire course of treatment immediately after application of the frame. Additionally, no physiotherapy sessions were needed to regain normal full activity. The main disadvantages of this procedure are that it is technically demanding, the need for imaging, its relatively high expense, depression may occur in some patients, and the absolute necessity of adequate care of the frame. From our point of view, immediate weight-bearing and the fact that the patient could go back to school were adequate justification for this procedure. Furthermore, a reduction in hospital stay and time needed for physiotherapy are also important considerations.

## Conclusions

This case report provides satisfactory evidence that LIPUS and an Ilizarov ring fixator are successful for managing diaphyseal fractures in Alagille syndrome. Some reports have described that although internal fixation is better tolerated by patients with lower morbidity and better (although delayed) mobility, it is associated with some complications, such as delayed weight-bearing, implant failure, need for further surgery, and less mobility in the knee and ankle. Although technically demanding, the procedure reported here is a reliable and efficient method of treating simple femoral shaft fractures in Alagille syndrome because it resulted in adequate healing time, immediate ambulation and weight-bearing, excellent ankle and knee motion, and no complications. Based on the final clinical and radiographic outcomes, this technique proved to be adequate for managing a simple diaphyseal femoral fracture in Alagille syndrome. LIPUS treatment is an effective, non-invasive adjuvant method to enhance callus maturation in fracture healing. Using this treatment, the healing time and duration of external fixation can be reliably shortened.

## Consent

Written informed consent was obtained from the patient for publication of this Case Report and any accompanying images. A copy of the written consent is available for review by the Editor of this journal.

## Competing interests

The authors declare that they have no competing interests.

## Authors’ contributions

KN performed the surgery. YS helped with surgery and helped to draft the manuscript. SY assisted with surgery. YK, NM, and AN helped draft the manuscript. All authors read and approved the final manuscript.

## Pre-publication history

The pre-publication history for this paper can be accessed here:

http://www.biomedcentral.com/1471-2474/15/225/prepub
